# Getting it wrong most of the time? Comparing trialists’ choice of primary outcome with what patients and health professionals want

**DOI:** 10.1186/s13063-022-06348-z

**Published:** 2022-06-27

**Authors:** Shaun Treweek, Viviane Miyakoda, Dylan Burke, Frances Shiely

**Affiliations:** 1 Health Services Research Unit, University of Aberdeen, Aberdeen, UK; 2 Janssen-Cilag Farmacêutica Ltda, São Paulo, Brazil; 3 HRB Clinical Research Facility, Trial Forge, University College Cork, Cork, Ireland; 4 School of Public Health, University College Cork, Cork, Ireland

**Keywords:** Outcomes, Trial methodology, Clinical trials

## Abstract

**Background:**

Randomised trials support improved decision-making through the data they collect. One important piece of data is the primary outcome — so called because it is what the investigators decide is the most important. Secondary outcomes provide additional information to support decision-making. We were interested in knowing how important patients and healthcare professionals consider the outcomes (especially the primary outcome) measured in a selection of published trials.

**Methods:**

The work had three stages: (1) We identified a body of late-stage trials in two clinical areas, breast cancer management and nephrology. (2) We identified the primary and secondary outcomes for these trials. (3) We randomly ordered these outcomes and presented them to patients and healthcare professionals (with experience of the clinical area), and we asked them to rank the importance of the outcomes. They were not told which outcomes trial authors considered primary and secondary.

**Results:**

In our sample of 44 trials with 46 primary outcomes, 29 patients, one patient representative and 12 healthcare professionals together ranked the primary outcome as the most important outcome 13/46 times or 28%. Breast cancer patients and healthcare professionals considered the primary outcome to be the most important outcome for 8/21 primary outcomes chosen by trialists. For nephrology, the equivalent figure was 5/25. The primary outcome appeared in a respondent’s top 5 ranked outcomes 151/178 (85%) times for breast cancer and 225/259 (87%) times for nephrology even if the primary was not considered the most important outcome.

**Conclusions:**

The primary outcome in a trial is the most important piece of data collected. It is used to determine how many participants are required, and it is the main piece of information used to judge whether the intervention is effective or not. In our study, patients and healthcare professionals agreed with the choice of the primary outcome made by trial teams doing late-stage trials in breast cancer management and nephrology 28% of the time.

**Supplementary Information:**

The online version contains supplementary material available at 10.1186/s13063-022-06348-z.

## Background

Randomised trials are conducted to provide evidence to support better and more informed decisions about medicine and other healthcare initiatives. Trials support these decisions through the data they collect. How data are collected varies — it might be through a bespoke data collection process or through linkage to other data sources such as disease registries and electronic medical records. It could be a combination of the two. Regardless, if a trial dataset is silent on something important to decision-makers, then the trial will not meet its intended aim of supporting better and more informed decisions. Put simply, it has failed.

Trials can collect a lot of data, much of them (around 70%) outcome data [1]. Not all outcomes are created equal: participants, trial teams, the public, funders and other trial stakeholders are more interested in some than others. Trial teams themselves declare one outcome (or occasionally a few) to be the most important outcome and call it the primary outcome. The primary outcome generally drives the size of the trial [2] and future judgements as to whether the trial intervention is effective are largely framed around the primary outcome. All other outcomes are then, by definition, of less importance and are widely known as secondary outcomes.

The outcome choices made by trial teams have not always matched what decision-makers, patients especially, need to support their decisions [3]. For example, a review of 413 cardiovascular trials published in ten leading medical journals found that only 23% had a primary outcome ranked as important by patients, such as death, morbidity and health-related quality of life [4]. Composite outcomes were flagged as a particular problem because they often combine important and less important outcomes together, making interpretation difficult [4, 5]. Surrogate outcomes often appeal to trial teams because they can show change sooner (making a trial shorter) and may be easier and cheaper to measure. Using them is reasonable if there is a clear link between the surrogate and an outcome of known importance to decision-makers but they are also used where this link is doubtful. An analysis of 626 trials in a range of disease areas found that 109 (17%) used a surrogate primary outcome but only 38 (35%) also discussed its validity [6]. Missing data compounds these problems: a study including 143 systematic reviews of trials found that in 102 (71%) reviews there were missing data for key outcomes and 26 (18%) had primary outcome data from fewer than half of their participants [7].

Core outcome sets, an agreed minimum set of outcomes that should be collected for a particular type of trial, are an approach that helps to reduce these problems [5]. This is especially true where patients and public contributors are involved in the development of the set, as recommended by the Core Outcomes Measures in Effectiveness Trials (COMET) initiative [5]. Although core outcome sets do not rank the outcomes within a set, each outcome is known to be important because there has been a formal prioritisation process to select it. In other words, it both narrows the search for a primary outcome (why choose a primary outcome that is not in the core outcome set?) and forces trial teams to carefully justify the collection of outcomes not in the set.

Data collection represents work for participants, site staff and the central trial team. This work is only worthwhile if the information it provides is considered important by the people whose decisions the trial is intended to support. If the information is not considered important by these decision-makers, then the work spent getting it is an expensive form of research garnish, present but chiefly decorative.

With this in mind, the current study asked two simple questions:

1. How important do patients and healthcare professionals consider the outcomes measured in a selection of published trials?
2. Do patients and healthcare professionals select the trial primary outcome as the most important outcome?

The study was done as part of the Trial Forge initiative (www.trialforge.org) to improve trial efficiency and in collaboration with Ireland’s Health Research Board Trial Methodology Research Network.

## Methods

The work had three stages:

1. Identify a body of trials in one or more clinical areas that will provide the study sample
2. Identify the primary and secondary outcomes for the trials identified in #1
3. Present the trials and outcomes from #2 to patients and healthcare professionals with experience of the clinical area and ask them to rank the importance of the outcomes

These three stages were used in two related studies. The first study was done in breast cancer management and formed VM’s MSc dissertation project, which was supervised by ST. The second study was led by DB and FS and was done in nephrology. The choice of these clinical areas was based on convenience: we had interests and contacts in these clinical areas, which made stage 3 easier. The methods used for breast cancer and nephrology were almost, but not quite, the same, with the nephrology study learning from the experience of the breast cancer study. We highlight differences below when we describe each of the three stages.

### Stage 1 – Identify a body of trials

The eligibility criteria for trials were:

- The trial focused on the treatment of breast cancer or the management of side effects/consequences of the treatment, or the trial focused on the treatment and/or management of any nephrology-related illness some of which included dialysis patients, e.g. polycystic kidney disease, acute kidney injury, chronic kidney disease, progressive membranous nephropathy, diabetic kidney disease, end-stage renal disease, etc.
-  The trial was phase 3 or 4 (breast cancer), or phase 2, 3 or 4 (nephrology)
-  The trial could be industry- or academic-led
- The trial had to clearly report primary and secondary outcomes
- The trial results were published between 01/01/2015 and 31/12/2018 (breast cancer) and 01/01/2010 and 31/12/2019 (nephrology)

We made the pragmatic choice to limit our search to trials published in the *New England Journal of Medicine*, *The Lancet* and the *BMJ* for the breast cancer studies because these are key journals for publishing trials, including in breast cancer trials. For nephrology, we chose the *New England Journal of Medicine*, *The Lancet*, *The Journal of the American Medical Association*, *the Clinical Journal of the American Society of Nephrology, Kidney International* and *Nephrology Dialysis Transplantation* because these are key journals for publishing nephrology trials. Our journal choice meant all articles were written in English. There are relatively few randomised controlled trials conducted in nephrology [8], necessitating the broadening of the search to six journals, including phase 2 trials and having a wider timeframe than the breast cancer study. The search strategies for breast cancer and nephrology are given in Supplementary File 1. Abstracts were screened in duplicate by two of the authors (VM and ST for breast cancer; DB and FS for nephrology) and the list of potentially eligible studies was then agreed through discussion in these pairs.

We set ourselves a target sample size of 20 trials for breast cancer and 25 for nephrology. We wanted a sample that was large enough to say something meaningful but not so large that patients and healthcare professionals would be overwhelmed by the number of trials they were asked to review, particularly given the large number of outcomes listed for some trials. Additionally, as the breast cancer work was part of an MSc, the nephrology work was part of a 4-month work placement and all the work was done without dedicated funding, a sample of 20/25 trials per condition seemed a reasonable compromise between sample size and feasibility.

We anticipated that the list of potentially eligible studies identified by our searches would be greater than our target of 20/25 per condition, meaning we would need to make a selection. We did this by randomly selecting articles from the list of all eligible studies for each condition. If a selected study did not meet the eligibility criteria after full-text review, the study was removed, and a replacement study was randomly selected.

### Stage 2 — Identifying primary and secondary outcomes

The data extracted for each article are shown in Table 1. The trial outcomes explicitly called primary and secondary by the trial authors were extracted by VM (breast cancer) and DB (nephrology). Any other outcomes (e.g. those classified by the trial authors as exploratory) were not extracted. For breast cancer, the short outcome definitions mentioned in Table 1 were written by VM and ST and were included to try and make outcomes more understandable to patients and healthcare professionals when they were asked to review the trials in stage 3. For the nephrology study, the healthcare professionals received the outcomes as presented in the original trial and the patients received the short outcome version, which had been modified by FS, DB and a consultant nephrologist. The expertise of the consultant nephrologist ensured the outcome definitions were matched in meaning and minimised any impact of the varied wording. For both breast cancer and nephrology, all presentations included a description of the trial together with its primary and secondary outcomes. This was done in a structured way to be consistent across trials and, we hoped, to reduce participant burden. Supplementary File 2 is an example of how two trials (both breast cancer), their outcomes and the short outcome definitions (where needed) were presented to participants. The order in which outcomes were presented was random, which meant that the position of an outcome in the list held no significance.

**Table 1:** The data extracted for each included trial

Data extraction
Trial reference
Brief description of the intervention being tested (plain language)
Brief description of the study population
Primary outcome(s) including a short definition
Secondary outcome 1 (including a short definition)
Secondary outcome 2 (including a short definition)
Secondary outcome 3 (including a short definition)
Secondary outcome 4 (including a short definition)
Etc…

### Stage 3 – Presenting the trials and outcomes to patients and healthcare professionals

Our aim was to present the results of stage 2 to people who could represent the needs of ordinary patients and healthcare professionals when it came to decisions about breast cancer management or nephrology. We acknowledge that a relatively small group of patients and health professionals will not reflect the full range of lived experiences and treatment decisions that would have been ideal for the body of trials we had selected. Nevertheless, all would have made real breast cancer or nephrology treatment decisions for themselves or others. In the case of patients, they were likely to have lived experience of all or some of the outcomes we presented to them and probably more than the teams that designed the trials.

For breast cancer, the targeted stakeholders considered were:

- Medical and clinical oncologists
- Surgeons
- Radiologists
- Breast care nurses
- Representatives from cancer organisations
- Representatives from patient advocacy groups
- People who have, or have had, breast cancer

The equivalent list for nephrology was:

- Consultant nephrologists
- Registrar in nephrology
- Renal dietician
- Patients who attend an outpatient nephrology clinic

We created a participant information leaflet for the breast cancer study to send to the people we invited to stage 3 (Supplementary File 3), and we provided more information as needed by email from one of VM or ST. Invitees were identified through our personal networks (the UK and USA for ST, Brazil for VM; see Table 2) for both professional and public and patient contributors. We did not invite anyone from our own institutions. We stopped sending invitations when ten individuals had said they would take part; all those we asked agreed to take part. The presentation of trials and outcomes for the breast cancer study was done online using the free version of SurveyMonkey (https://www.surveymonkey.co.uk).

**Table 2:** Stakeholder’s panel composition

**Breast cancer management (10 individuals)**	
** Stakeholder’s category**	**Country**
Breast cancer surgeon representative from a Clinical Oncology Society	Brazil
Pharmacist representative from a Clinical Oncology Society	Brazil
Clinical oncologist × 2	Brazil
Breast cancer surgeon	UK
Breast cancer surgeon	USA
Radiologist and professor of breast imaging	UK
Person who has had treatment for breast cancer × 2	UK
Representative of a Cancer Patient Advocacy Group	Brazil
**Nephrology (32 individuals)**	
** Stakeholder’s category**	**Country**
Consultant nephrologist × 3	Ireland
Registrar in nephrology × 1	Ireland
Renal dietician × 1	Ireland
Patients who attend an outpatient nephrology clinic × 27	Ireland

For the nephrology study, we chose to involve health professionals and patients through a single route: the Renal Outpatient Clinic at Cork University Hospital. The consultant nephrologist, in the presence of FS, briefed the renal nurses and the consultants on the conduct of the study. For patients, we conducted a pilot study on patients at the outpatient clinic (*n* = 3) having received some early feedback from the healthcare professionals that it was a challenging and time-consuming task. All three patients only partially completed the task due to the length of the questionnaire. Thus, for the main study, we divided the 25 trials, randomly, into 5 separate batches, A, B, C, D and E, each with five trials. For the patient questionnaire, FS and a colleague (EM) attended five different outpatient clinics over a period of 3 weeks and approached patients in the waiting room about participation in the study. All patient participants received a patient information leaflet and signed an informed consent form agreeing to participate in the study (Supplementary File 4). They received a hard copy of either batch A, B, C, D or E and completed it in the presence of either EM or FS who could then answer any questions participants had. We targeted 25 trials for completion by the patients. Healthcare professionals received the outcomes for the 25 trials by email in Microsoft Word and each also signed an informed consent form.

All responses were anonymous and no personal data were collected. Each person was asked to rank the five most important outcomes (breast cancer) or rank all outcomes (nephrology). Free-text comments could also be left, which could be used to list outcomes considered important, but which were not collected in the trial. Any trial could be skipped to be answered later, or just left blank.

### Analysis

Analysis was simple — we calculated median ranking and an inter-quartile range to tell us how important patients and health professionals thought the outcomes were. For breast cancer, we only asked people to choose their top 5, which meant that other outcomes were classed as unranked. We had full ranking information for nephrology.

## Results

### Stages 1 and 2

For breast cancer, we had 64 eligible trials from which we randomly selected 20. For nephrology, we had 32 eligible trials from which we randomly selected 25. However, there were doubts about the suitability of one of the nephrology trials because it used a composite primary outcome and, after discussion between FS and ST, that trial was removed. This meant the nephrology sample was 24 trials. A summary of all 44 included trials is given in Supplementary File 5. The breast cancer trials included 128 outcomes in total of which 21 were primary outcomes; the nephrology trials included 145 outcomes of which 25 were primary outcomes.

### Stage 3

Table 2 shows the stakeholders who ranked outcomes for the breast cancer (two patients, one patient representative and seven health professionals) and nephrology trials (27 patients, five health professionals). The email with the link to the breast cancer trials and outcomes was sent on 31 May 2019 and we had responses from all members by 15 July 2019, which is when we closed the SurveyMonkey system. The nephrology pilot study was conducted in February 2020, but the main data collection was delayed due to the COVID-19 pandemic. The patient surveys were collected in June–July 2020 and the healthcare professional surveys were collected in October and November 2020.

Median (with range) rankings of the primary outcome and the highest ranked outcome for each of the 20 breast cancer management trials is given in Table 3. Trial 13 had two primary outcomes so appears twice. The equivalent data for the 24 nephrology trials are given in Table 4; here trial 16 had two primary outcomes so appears twice. The full datasets for breast cancer and nephrology trials showing rankings for all outcomes are available at https://osf.io/xkad6/.

**Table 3:** Summary of the primary outcomes, weighted medians and outcomes with highest ranked weighted medians for the 21 breast cancer trial primary outcomes. The eight trials marked [**Primary outcome match**] are those where the primary outcome was either considered most important by the panel or was among those considered equally most important by the panel

Trial ID	Primary outcome	Number of responses to trial	Number of outcomes	Median ranking of primary [range]	Number of times primary was within respondents’ top 5 outcomes	Outcome(s) with highest ranked median	Median ranking of highest placed outcome [range]
**Trial 1**	Locally investigator-assessed progression-free survival	10	6	3 [2–unranked]	9	Overall survival	1 [1–unranked]
*HCPs*		*7*		*4 [3–unranked]*	*6*	*Overall survival*	*1 [1–unranked]*
*Patients*		*3*		*2* *[2-2]*	*3*	*Overall survival*	*1 [1-2]*
**Trial 2**	Survival without distant metastasis at 5 years (time from trial randomisation until the first distant metastatic recurrence, or death from any cause)	11	4	3 [1-5]	11	Overall survival (time from trial randomisation until death from any cause) Disease-free survival (time from trial randomisation until first disease progression or death from any cause)	2 [1-5] 2 [1-5]
*HCPs*		*8*		*3.5 *[1-5]	*8*	*Overall survival (time from trial randomisation until death from any cause)*	*2 [1-5]*
*Patients*		*3*		*2 *[1-3]	*3*	*Disease-free survival (time from trial randomisation until first disease progression or death from any cause)*	*1 [1,2]*
**Trial 3**	Invasive disease-free survival at 2-year follow-up. Defined as the time from randomisation to any of the following: invasive tumour recurrence (same side as the original tumour), invasive breast cancer (opposite side), local or wider invasive recurrence, more distant recurrence, death from any cause	10	7	2.5 [1–unranked]	4	Overall survival (time from randomisation to death)	2 [1–unranked]
*HCPs*		*7*		*3 [2–unranked]*	*6*	*Overall survival (time from randomisation to death)*	*2 [1-5]*
*Patients*		*3*		*1 [1–unranked]*	*1*	*Invasive disease-free survival at 2-year follow-up. Defined as the time from randomisation to any of the following: invasive tumour recurrence (same side as the original tumour), invasive breast cancer (opposite side), local or wider invasive recurrence, more distant recurrence and death from any cause* *Cumulative incidence of central nervous system (CNS) cancer recurrences (time from randomisation to CNS recurrence as first distant recurrence)* *Safety*	*1 [1–unranked]* *1 [1–unranked]* *1 [1–unranked]*
**Trial 4**	Overall survival (time from randomisation to death from any cause)	9	4	2 [1-5]	9	Health-related quality of life	1 [1.5–unranked]
*HCPs*		*6*		*2.5 [1-5]*	*6*	*Health-related quality of life*	*1.5 [1-4]*
*Patients*		*3*		*1 [1-2]*	*3*	*Overall survival (time from randomisation to death from any cause)*	*1 [1-2]*
**Trial 5**	Overall survival (time from randomisation to death from any cause)	9	5	2 [1–unranked]	6	Difference between treatment groups in the change in systolic automated office blood pressure from baseline to week 12	2 [1-7]
*HCPs*		*6*		*1 [1–unranked]*	*5*	*Difference between treatment groups in the change in systolic automated office blood pressure from baseline to week 12*	*2 [1-5]*
*Patients*		*3*		*3 [3–unranked]*	*1*	*Cumulative incidence of regional recurrence and distant metastasis (i.e. that has spread)* *Survival time*	*1 [1-1]* *1 [1-2]*
**Trial 6**	Breast cancer-free interval (time from randomisation to any breast cancer event censored for deaths)	9	12	3 [1–unranked]	7	Vaginal symptoms (vaginal dryness and pain with intercourse) Disease-free survival (time to any recurrence excluding lobular carcinoma in situ, second primary cancer and death from any cause) Quality of life	2 [1–unranked] 2 [1–unranked] 2 [2-5]
*HCPs*		*6*		*3.5 [2–unranked]*	*4*	*Quality of life*	*2 [2-4]*
*Patients*		*3*		*2 [1-3]*	*3*	*Vaginal symptoms (vaginal dryness and pain with intercourse)* *Disease-free survival (time to any recurrence excluding lobular carcinoma *in situ*, second primary cancer and death from any cause)* *Contralateral breast cancer* *Osteoporotic fractures* *Vasomotor symptoms* *Sexual functioning*	*1 [1–unranked]* *1 [1-2]* *1 [1–unranked]* *1 [1–unranked]* *1 [1–unranked]* *1 [1–unranked]*
**Trial 7**	Pathological complete response of the primary tumour in the breast (absence of histological evidence of invasive tumour cells in the breast sample removed at surgery)	8	5	2 [1-5]	8	Disease-free survival (time from randomisation to disease recurrence, or death)	1 [1–unranked]
*HCPs*		*5*		*2 [1-5]*	*5*	*Pathological complete response of the primary tumour in the breast (absence of histological evidence of invasive tumour cells in the breast sample removed at surgery)*	*2 [1-5]*
*Patients*		*3*		*2 [2-2]*	*5*	*Disease-free survival (time from randomisation to disease recurrence, or death)*	*1 [1-1]*
Trial 8 [**Primary outcome match**]	Disease-free survival (time from randomisation to the date of one of the following (whichever came first): local recurrence, distant metastases — i.e. cancer that has spread, contralateral or ipsilateral breast tumor (excluding ductal carcinoma in situ — i.e. cancer that has not spread), second primary malignancy, death from any cause, loss to follow-up or end of the study	9	3	2 [1-4]	9	Disease-free survival (time from randomisation to the date of one of the following (whichever came first): local recurrence, distant metastases — i.e. cancer that has spread, contralateral or ipsilateral breast tumor (excluding ductal carcinoma in situ — i.e. cancer that has not spread), second primary malignancy, death from any cause, loss to follow-up or end of the study Overall survival (time from randomisation to the date of death from any cause, loss to follow-up or the end of study)	2 [1-4] 2 [1-5]
*HCPs*		*6*		*2 [1-4]*	*6*	*Overall survival (time from randomisation to the date of death from any cause, loss to follow-up or the end of study)*	*1.5 [1-5]*
*Patients*		*3*		*1 [1-2]*	*3*	*Disease-free survival (time from randomisation to the date of one of the following (whichever came first): local recurrence, distant metastases — i.e. cancer that has spread, contralateral or ipsilateral breast tumor (excluding ductal carcinoma *in situ* — i.e. cancer that has not spread), second primary malignancy, death from any cause, loss to follow-up or end of the study*	*1 [1-2]*
**Trial 9**	Time-to-event analysis of the rate of survival free from invasive cancer (first event of recurrence of one of the following: ipsilateral breast tumour, local recurrence, regional recurrence, distant recurrence, contralateral second primary invasive cancer, second primary non-breast invasive cancer (excluding non-melanoma skin cancer), or death without evidence of recurrence))	8	4	3 [1–unranked]	7	Overall survival rate (proportion of patients who did not die from any cause) Freedom from any recurrence (first recurrence of breast cancer at any site or death with recurrence)	2 [1–unranked] 2 [1–unranked]
*HCPs*		*6*		*3 [1–unranked]*	*5*	*Overall survival rate (proportion of patients who did not die from any cause)* *Freedom from any recurrence (first recurrence of breast cancer at any site or death with recurrence)*	*2 [1–unranked]* *2 [1–unranked]*
*Patients*		*2*		*3 [3-3]*	*2*	*Freedom from any recurrence (first recurrence of breast cancer at any site or death with recurrence)*	*2 [2-2]*
Trial 10 [**Primary outcome match**]	Progression-free survival (determined based on investigator’s assessments according to response evaluation criteria in solid tumours, or surgery or radiotherapy for worsening of disease, or death from any cause)	8	10	2 [1–unranked]	7	Clinical benefit rate (best overall response of complete response, partial response, or stable disease ≥ 24 weeks) Progression-free survival (determined based on investigator’s assessments according to response evaluation criteria in solid tumours, or surgery or radiotherapy for worsening of disease, or death from any cause)	2 [1–unranked] 2 [1–unranked]
*HCPs*		*5*		*2.5 [2–unranked]*	*4*	*Objective response rate (best overall response of either complete response or partial response in patients with measurable disease at baseline)*	*2 [1–unranked]*
*Patients*		*3*		*1 [1-2]*	*3*	*Progression-free survival (determined based on investigator’s assessments according to response evaluation criteria in solid tumours, or surgery or radiotherapy for worsening of disease, or death from any cause)* *Health-related quality of life*	*1 [1-2]* *1 [1–unranked]*
Trial 11 [**Primary outcome match**]	All recurrence (development of histologically confirmed breast cancer both invasive and new or recurrent ductal carcinoma in situ (DCIS))	9	8	1 [1–unranked]	7	All recurrence (development of histologically confirmed breast cancer both invasive and new or recurrent ductal carcinoma in situ (DCIS))	1 [1–unranked]
*HCPs*		*6*		*1.5 [1–unranked]*	*4*	*All recurrence (development of histologically confirmed breast cancer both invasive and new or recurrent ductal carcinoma *in situ* (DCIS))*	*1.5 [1–unranked]*
*Patients*		*3*		*1 [1-1]*	*3*	*All recurrence (development of histologically confirmed breast cancer both invasive and new or recurrent ductal carcinoma *in situ* (DCIS))*	*1 [1-1]*
**Trial 12**	Overall survival (time from randomisation to death from any cause)	9	8	2 [1-5]	9	Progression-free survival (time from randomisation to disease progression or death from any cause)	1 [1-5]
*HCPs*		*6*		*2 [1-5]*	*6*	*Progression-free survival (time from randomisation to disease progression or death from any cause)*	*1.5 [1-5]*
*Patients*		*3*		*2 [2-2]*	*3*	*Progression-free survival (time from randomisation to disease progression or death from any cause)*	*1 [1-1]*
**Trial 13: primary 1**	Investigator-assessed progression-free survival (time from randomisation to first documented disease progression according to RECIST or death from any cause)	8	10	2.5 [1–unranked]	6	Overall survival (interval from randomisation to death from any cause)	1.5 [1-5]
*HCPs*		*6*		*3 [2–unranked]*	*4*	*Overall survival (interval from randomisation to death from any cause)*	*1.5 *[1–5]
*Patients*		*2*		*1.5 [1-2]*	*2*	*Investigator-assessed progression-free survival (time from randomisation to first documented disease progression according to RECIST or death from any cause)* *Overall survival (interval from randomisation to death from any cause)*	*1.5 [1-2]* *1.5 [1-2]*
Trial 13: primary 2 [**Primary outcome match**]	Overall survival (interval from randomisation to death from any cause)	8	10	1.5 [1-5]	*8*	Overall survival (interval from randomisation to death from any cause)	1.5 [1-5]
*HCPs*		*6*		*1.5 [1-5]*	*6*	*Overall survival (interval from randomisation to death from any cause)*	*1.5 [1-5]*
*Patients*		*2*		*1.5 [1-2]*	*2*	*Investigator-assessed progression-free survival (time from randomisation to first documented disease progression according to RECIST or death from any cause)* *Overall survival (interval from randomisation to death from any cause)*	*1.5 [1-2]* *1.5 [1-2]*
**Trial 14**	5-year disease-free survival (date of randomisation to the date of first relapse, to the date of death in women dying without relapse or to the date of censor in women alive and relapse-free)	8	7	1.5 [1–unranked]	4	10-year disease-free survival	1 [1–unranked]
*HCPs*		*6*		*3 [2-3]*	*5*	*10-year disease-free survival*	*1 [1–unranked]*
*Patients*		*2*		*4 [3-7]*	*4*	*5-year disease-free survival (date of randomisation to the date of first relapse, to the date of death in women dying without relapse or to the date of censor in women alive and relapse-free)*	*1 [1–unranked]*
**Trial 15**	Pathological complete response (absence of invasive breast cancer in the breast and axillary lymph nodes, after neoadjuvant chemotherapy)	8	4	2.5 [1-5]	8	Disease-free survival	1 [1–unranked]
*HCPs*		*6*		*2 [1-5]*	*6*	*Disease-free survival*	*1.5 [1–unranked]*
*Patients*		*2*		*3 [3-3]*	*2*	*Disease-free survival*	*1 *[1]
Trial 16 [**Primary outcome match**]	Progression-free survival (time from trial randomisation until the first progression of the cancer, or death due to any cause, whichever occurred first)	8	9	2 [1–unranked]	7	Overall survival (time from randomisation to the date of death due to any cause) Quality of life Progression-free survival (time from trial randomisation until the first progression of the cancer, or death due to any cause, whichever occurred first) Clinical benefit (proportion of patients whose final response to treatment is judged to be (a) complete response or (b) partial response, or who have stable cancer for ≥ 24 weeks)	2 [1–unranked] 2 [1–unranked] 2 [1–unranked] 2 [1–unranked]
*HCPs*		*6*		*2 [1–unranked]*	*5*	1*) Overall survival (time from randomisation to the date of death due to any cause)* *2) Clinical benefit (proportion of patients whose final response to treatment is judged to be a) complete response or b) partial response, or who have stable cancer for* ≥ *24 weeks)*	*1 [1–unranked]* *1 [1–unranked]*
*Patients*		*2*		*2 [1-3]*	*2*	*1) Overall survival (time from randomisation to the date of death due to any cause)* *2) Progression-free survival (time from trial randomisation until the first progression of the cancer, or death due to any cause, whichever occurred first)*	*2 [2-2]* *2 [1-3]*
Trial 17 [**Primary outcome match**]	Occurrence of any type of breast cancer (including ductal carcinoma in situ — i.e. cancer that has not spread)	8	4	1.5 [1-5]]	8	Occurrence of any type of breast cancer (including ductal carcinoma in situ — i.e. cancer that has not spread)	1.5 [1-5]
*HCPs*		*6*		*1.5 [1-5]*	*6*	*Occurrence of any type of breast cancer (including ductal carcinoma *in situ* — i.e. cancer that has not spread)*	*1.5 [1-5]*
*Patients*		*2*		*1.5 [1-2]*	*2*	*Occurrence of any type of breast cancer (including ductal carcinoma *in situ* — i.e. cancer that has not spread)* *Occurrence of invasive oestrogen receptor-positive breast cancer*	*1.5 [1-2]* *1.5 [1-2]*
Trial 18 [**Primary outcome match**]	Invasive disease-free survival (time from randomisation until the date of the first occurrence of one of the following events: recurrence of ipsilateral invasive breast tumor, recurrence of ipsilateral locoregional invasive breast cancer, contralateral invasive breast cancer, a distant disease recurrence or death from any cause)	8	6	2 [1–unranked]	5	Invasive disease-free survival (time from randomisation until the date of the first occurrence of one of the following events: recurrence of ipsilateral invasive breast tumor, recurrence of ipsilateral locoregional invasive breast cancer, contralateral invasive breast cancer, a distant disease recurrence or death from any cause) Overall survival Safety Disease-free survival (including noninvasive breast cancers)	2 [1–unranked] 2 [1–unranked] 2 [2–unranked] 2 [1–unranked]
*HCPs*		*6*		*2.5 [1–unranked]*	*4*	*Overall survival*	*1 [1-5]*
*Patients*		*2*		*1 [1-1]*	*1*	*Invasive disease-free survival (time from randomisation until the date of the first occurrence of one of the following events: recurrence of ipsilateral invasive breast tumor, recurrence of ipsilateral locoregional invasive breast cancer, contralateral invasive breast cancer, a distant disease recurrence or death from any cause)*	*1 [1-1]*
Trial 19 [**Primary outcome match**]	Investigator-assessed progression-free survival (time from randomisation to radiologically confirmed disease progression according to RECIST, version 1.1, or death during the study)	7	8	2 [1-4]	7	Clinical benefit response (defined as a confirmed complete response, a partial response or stable disease for ≥ 24 weeks) Investigator-assessed progression-free survival (time from randomisation to radiologically confirmed disease progression according to RECIST, version 1.1, or death during the study)	2 [1–unranked] 2 [1-4]
*HCPs*		*5*		*3 [1-4]*	*5*	*Clinical benefit response (defined as a confirmed complete response, a partial response or stable disease for* ≥ *24 weeks)* *Overall survival objective response (defined as confirmed complete response or partial response)* *Patient-reported outcomes (assessed by health-related quality-of-life scores)*	*2 [2–unranked]* *2 [1–unranked]* *2 [2-3]*
*Patients*		*2*		*1.5 [1-2]*	*2*	*Investigator-assessed progression-free survival (time from randomisation to radiologically confirmed disease progression according to RECIST, version 1.1, or death during the study)*	*1.5 *[1-2]
**Trial 20**	Overall survival (time from randomisation to the date of death from any cause)	8	4	2 [1–unranked]	7	Disease-free survival (time from randomisation to the first date of local recurrence, regional recurrence, distant recurrence, second breast cancer or death from any cause, whichever occurred first)	1 [1–unranked]
*HCPs*		*6*		*2 [1–unranked]*	*5*	*Disease-free survival (time from randomisation to the first date of local recurrence, regional recurrence, distant recurrence, second breast cancer or death from any cause, whichever occurred first)* *Overall survival (time from randomisation to the date of death from any cause)* *Distant disease-free survival (time from randomisation to the first date of distant disease or death from any cause, whichever occurred first)*	*2 [1–unranked]* *2 [1–unranked]* *2 [1-5]*
*Patients*		*2*		*3.5 [3-4]*	*2*	*Disease-free survival (time from randomisation to the first date of local recurrence, regional recurrence, distant recurrence, second breast cancer or death from any cause, whichever occurred first)*	*1 [1-1]*

**Table 4:** Summary of the primary outcomes, weighted medians and outcomes with highest ranked weighted medians for the 25 nephrology trial primary outcomes. The six trials marked [**Primary outcome match**] are those where the primary outcome was either considered most important by the panel or was among those considered equally most important by the panel

Trial ID	Primary outcome	Number of responses to trial	Number of outcomes	Median ranking of primary [range]	Number of times primary was within respondents’ top 5 outcomes	Outcome(s) with highest ranked median	Median ranking of highest placed outcome [range]
Trial 1 [**Primary outcome match**]	End-stage renal disease or death from cardiovascular causes	11	4	1 [1-4]	11	End-stage renal disease or death from cardiovascular causes	1 [1-4]
* HCPs*		*5*		*1 [1-3]*	*4*		*1 [1-3]*
* Patients*		*6*		*1.5 [1-4]*	*6*		*1.5 [1-4]*
**Trial 2**	Mean change from the baseline haemoglobin level to the mean level during the evaluation period	11	3	2 [1-3]	11	Mean change from the baseline haemoglobin level to the mean level during the evaluation period Proportion of patients in whom there was a response in haemoglobin levels Proportion of patients receiving a transfusion	2 [1-3] 2 [1-3] 2 [1-3]
* HCPs*		*6*		*2 [1-3]*	*6*	*Mean change from the baseline haemoglobin level to the mean level during the evaluation period*	*1 [1-3]*
*Patients*		*5*		*1 [1-3]*	*4*	*1) Mean change from the baseline haemoglobin level to the mean level during the evaluation period* *2) Proportion of patients in whom there was a response in haemoglobin levels 3) Proportion of patients receiving a transfusion*	*2 [1-3]* *2 [1-3]* *2 [1-3]*
**Trial 3**	Occurrence of a major cardiovascular or renal event	10	9	4 *[1-9]*	8	1) Occurrence of a major cardiovascular or renal event 2) Need for dialysis or transplantation 3) Worsening of kidney function	4 *[1-9]* 4 *[1-6]* 4 *[1-9]*
* HCPs*		*5*		*3 [1-5]*	*5*	*All-cause mortality*	*1 [1-6]*
* Patients*		*5*		*4 [3-9]*	*3*	*Need for dialysis or transplantation*	*1 [1-6]*
**Trial 4**	Difference between treatment groups in the proportion of patients remaining on spironolactone at week 12	11	7	5 *[1-7]*	6	Difference between treatment groups in the change in systolic automated office blood pressure from baseline to week 12	2 *[1-7]*
* HCPs*		*5*		*5 [1-6]*	*3*	*Difference between treatment groups in the change in systolic automated office blood pressure from baseline to week 12*	*2 [1-5]*
* Patients*		*6*		*5.5 [2-7]*	*3*	*Patient-reported outcomes as measured by the EQ-5D-5L questionnaire*	*1.5 [1-7]*
**Trial 5**	Independence from dialysis at 90 days after random allocation to groups	10	5	3.5 [1-5]	10	Overall survival at the end of the study	1 [1-5]
* HCPs*		*5*		*3 [1-4]*	*5*	*Overall survival at the end of the study*	*1 [1-5]*
* Patients*		*5*		*4 [1-5]*	*5*	*Overall survival at the end of the study*	*1 [1-5]*
Trial 6 [**Primary outcome match**]	A composite of death from any cause by 28 days after randomisation and the presence of at least one organ failure at 7 days after randomization	10	7	1 [1-3]	10	A composite of death from any cause by 28 days after randomisation and the presence of at least one organ failure at 7 days after randomization	1 [1-3]
* HCPs*		*5*		*1 [1-1]*	*5*	*A composite of death from any cause by 28 days after randomisation and the presence of at least one organ failure at 7 days after randomization*	*1 [1-1]*
* Patients*		*5*		*2 [1-3]*	*5*	*A composite of death from any cause by 28 days after randomisation and the presence of at least one organ failure at 7 days after randomization*	*2 [1-3]*
**Trial 7**	A ranking of clinical outcomes, maximum relative change in creatinine, dialysis, and death, within 7 days of randomization	10	6	2.5 [1-5]	10	Death	2 [1-6]
* HCPs*		*5*		*2 [2-5]*	*5*	*Death*	*1 [1-4]*
* Patients*		*5*		*3 [1-5]*	*5*	*Progression to higher stages of acute kidney injury*	*2 [1-6]*
Trial 8 [**Primary outcome match**]	A further 20% decline in excretory renal function from baseline readings	10	3	1 [1-2]	10	A further 20% decline in excretory renal function from baseline readings	1 [1-2]
* HCPs*		*5*		*1 [1-2]*	*5*	*A further 20% decline in excretory renal function from baseline readings*	*1 [1-2]*
* Patients*		*5*		*1 [1-1]*	*5*	*A further 20% decline in excretory renal function from baseline readings*	*1 [1-1]*
**Trial 9**	Relapse-free survival based on the period of time until the first relapse	10	3	2 [1-3]	10	Relapse-free survival based on the period of time until the first relapse Probability of progression-free survival based on the time until the progression to FRNS, SDNS or SRN Relapse rate	2 [1-3] 2 [1-3] 2 [1-3]
* HCPs*		*5*		*2 [1-3]*	*5*	*Probability of progression-free survival based on the time until the progression to FRNS, SDNS or SRN*	*1 [1-3]*
* Patients*		*5*		*1 [1-3]*	*5*	*Relapse-free survival based on the period of time until the first relapse*	*1 [1-3]*
**Trial 10**	The change in UACR from baseline to the end of treatment	10	10	4 [2-9]	7	Proportion of participants in remission defined as a reversal of UACR to normoalbuminuria	3 [1-8]
* HCPs*		*5*		*4 [2-6]*	*4*	*Proportion of participants in remission defined as a reversal of UACR to normoalbuminuria* *Proportion of participants who sustained remission after treatment*	*2 [1-8]* *2 [1-5]*
* Patients*		*5*		*5 [2-9]*	*3*	*Plasma aldosterone concentration*	*2 [2-7]*
**Trial 11**	The change in log-transformed urine albumin-to-creatinine ratio from baseline to the end of treatment	10	9	3.5 [1-8]	9	eGFR The remission rate from early-stage nephropathy to prenephropathy stage at the end of treatment	2 [1-6] 2 [1-6]
* HCPs*		*5*		*3 [1-4]*	*5*	*The progression rate from early-stage nephropathy to overt nephropathy during the treatment period*	*2 [1-3]*
* Patients*		*5*		*5 [3-8]*	*4*	*eGFR*	*1 [1-2]*
Trial 12 [**Primary outcome match**]	Percentage of patients with treatment success	10	5	1 [1-5]	10	Percentage of patients with treatment success	1 [1-5]
* HCPs*		*5*		*1 [1-5]*	*5*	*Percentage of patients with treatment success*	*1 [1-5]*
* Patients*		*5*		*1 [1-5]*	*5*	*Percentage of patients with treatment success*	*1 *[1–5]
**Trial 13**	Change in cGFR from baseline to month 12	10	7	3 [2-7]	9	Patient and graft survival	1 [1-4]
* HCPs*		*5*		*3 [2-3]*	*5*	*Patient and graft survival*	*1 [1-1]*
* Patients*		*5*		*4 [3-7]*	*4*	*Incidence of AR*	*1 [1-4]*
**Trial 14**	The rate of haemodialysis independence at 3 months after randomization	10	10	7 [1-10]	3	Overall survival	1.5 [1-7]
* HCPs*		*5*		*7 [1-8]*		*Overall survival*	*1 [1-5]*
* Patients*		*5*		*8 [4-10]*		*Overall survival*	*2 [1-7]*
Trial 15: primary 1 [**Primary outcome match**]	Loss of kidney function at 1 year	12	2	1 [1-2]	12	Loss of kidney function at 1 year	1 [1-2]
* HCPs*		*5*		*1 [1-1]*	*5*	*Loss of kidney function at 1 year*	*1 [1-1]*
* Patients*		*7*		*2 [1-2]*	*7*	*Acute kidney injury within 30 days of surgery*	*1 [1-2]*
**Trial 15: primary 2**	Acute kidney injury within 30 days of surgery	12	2	*2 [1-2]*	12	Loss of kidney function at 1 year	1 [1-2]
* HCPs*		*5*		*2 [2-2]*	*5*	*Loss of kidney function at 1 year*	*1 [1-1]*
* Patients*		*7*		*1 [1-2]*	*7*	*Acute kidney injury within 30 days of surgery*	*1 [1-2]*
**Trial 16**	eGFR over time	12	8	3 [1-8]	10	Blood pressure control	2.5 [1-8]
* HCPs*		5		4 [1-8]	4	Blood pressure control	2 [1-3]
* Patients*		7		3 [1-8]	6	Early recognition and diagnosis of CKD	2 [1-6]
**Trial 17**	Change from baseline to 24 weeks in UACR in the first morning urine sample	11	8	4 [1-7]	9	Serum cystatin C-derived eGFR	3 [1-8]
* HCPs*		*5*		*1 [1-5]*	*5*	*Change from baseline to 24 weeks in UACR in the first morning urine sample*	*1 [1-5]*
* Patients*		*6*		*4.5 [2-7]*	*4*	*24-h total urine albumin excretion*	*2.5 [1-4]*
**Trial 18**	Relative change in serum phosphate concentrations from baseline to end of treatment	12	3	1.5 [1-2]	12	Relative change in serum phosphate concentrations from baseline to end of treatment	1.5 [1-2]
* HCPs*		5		1 [1-2]	5	Relative change in serum phosphate concentrations from baseline to end of treatment	1 [1-2]
* Patients*		7		2 [1-2]	7	Changes in corrected PTH concentrations from baseline to end of treatment Relative change in serum phosphate concentrations from baseline to end of treatment	2 [1-3] 2 [1-2]
**Trial 19**	Mean change in Hgb from baseline to end of treatment	12	4	1.5 [1-4]	12	Mean change in Hgb from baseline to end of treatment	1.5 [1-4]
* HCPs*		5		1 [1-1]	5	Mean change in Hgb from baseline to end of treatment	1 [1-1]
* Patients*		7		3 [1-4]	7	Mean intradialytic change from pre-haemodialysis to post-haemodialysis in serum iron, unsaturated iron-binding capacity (UIBC) and TSAT Mean change in Hgb from baseline every 4 weeks	2 [1-4] 2 [1-4]
**Trial 20**	Measured GFR at 12 months	9	10	4 [1-9]	6	Rate of change of eGFR calculated from creatinine values at 0, 1, 3, 6, 9 and 12 months	1 [1-5]
* HCPs*		5		4 [1-9]	4	Rate of change of eGFR calculated from creatinine values at 0, 1, 3, 6, 9 and 12 months	2 [1-5]
* Patients*		4		5 [3-9]	2	Rate of change of eGFR calculated from creatinine values at 0, 1, 3, 6, 9 and 12 months	1 [1-1]
**Trial 21**	Left ventricular mass index	9	10	5 [1-7]	5	All-cause mortality and hospitalization for CV events	1 [1-2]
* HCPs*				5 [4-7]	3	All-cause mortality and hospitalization for CV events	1 [1-2]
* Patients*				5 [1-7]	2	1) 2) All-cause mortality and hospitalization for CV events Exercise capacity: New York Heart Association functional class, 6-min walk test	2 [1-2] 2 [1-7]
**Trial 22**	Percentage of subjects achieving an increase in Hb of ≥ 1.0 g/dL at any study point between baseline and End of Study or introduction or dose increase of ESA, blood transfusion or use of iron outside of protocol	9	5	3 [2-5]	5	1) 2) Mean change in Hb from baseline to end of week 6 (day 42) and end of week 8 (day 56)/end of study Percent of subjects achieving a clinical response	2 [1-5] 2 [1-5]
* HCPs*		*5*		*3 [2-4]*	*5*	*Percent of subjects achieving a clinical response*	*1 [1-3]*
* Patients*		*4*		*3.5 [3-5]*	*4*	*Mean change from baseline to highest Hb*	*1 [1-4]*
**Trial 23**	Rate of s-K + decline in the first 48 h, using all post-baseline s-K + data	9	5	2 [1-3]	9	Rate of s-K + decline in the first 48 h, using all postbaseline s-K + data Kidney function parameters	2 [1-3] 2 [1-4]
* HCPs*		*5*		*2 [1-3]*	*5*	*Rate of s-K* + *decline in the first 48 h, using all postbaseline s-K* + *data* *Changes in s-K* + *at various time points after start of treatment*	*2 [1-3]* *2 [1-3]*
* Patients*		*4*		*3 [2-3]*	*4*	*Kidney function parameters*	*1 [1-1]*
**Trial 24**	Proportion of CRR (complete renal remission) at 24 weeks	9	2	2 [1-2]	9	CRR rate at 48 weeks	1 [1-2]
* HCPs*		*5*		*2 [1-2]*	*5*	*CRR rate at 48 weeks*	*1 [1-2]*
* Patients*		*4*		*1 [1-2]*	*4*	*Proportion of CRR (complete renal remission) at 24 weeks*	*1 [1-2]*

Our two most important results:

1. *Breast cancer* — patients/patient representative and health professionals considered the primary outcome to be the most important outcome for 8/21 primary outcomes
2. *Nephrology* — patients and health professionals considered the primary outcome to be the most important outcome for 5/25 primary outcomes

These matches are highlighted in Tables 3 and 4, respectively. The nephrology trials were a mixture of nine phase 3 trials (one of which had two primary outcomes), 11 phase 2 trials and two trials of uncertain phase but which were definitely not phase 1 (see Supplementary File 5). All five of the cases where patients and health professionals considered the primary outcome to be the most important outcome were phase 3 trials.

Moving to other results, the primary outcome appeared in a respondent’s top 5 ranked outcomes 151/178 (85%) times for breast cancer and 225/259 (87%) times for nephrology even if the primary was not considered the most important outcome. Tables 3 and 4 also present data separately for healthcare professionals and patients/patient representative and the results are different for the two trial types. For breast cancer trials, patients/patient representative tended to rank the primary outcome higher (11/21 primary outcomes) than healthcare professionals (4/21). For nephrology trials, the reverse was true: healthcare professionals ranked the primary higher 16/25 times compared to 6/25 for patients.

We had two free-text comments from patients and 15 from healthcare professionals for the breast cancer trials; the equivalent figures for nephrology were two and 23. One patient commented that the medical jargon for one trial was hard to understand, and the other comment described the difficulty of making treatment decisions more generally. Health professionals tended to comment about being unsure of the study setting, which would, or might, influence their ranking decisions. They often then gave the assumptions they had made regarding setting when making their choices. Several additional outcomes were suggested (e.g. quality of life) but only for some trials and then not by all respondents. All of the nephrology comments suggested additional outcomes (44 outcome suggestions in total), especially quality of life (suggested six times), death (ten times) and adverse events (ten times). All comments are available at https://osf.io/xkad6/ for both breast cancer and nephrology.

Finally, the range of rankings given for an outcome by patients/patient representative and healthcare professionals could be wide. This included the primary outcome; it was not uncommon for some people to rank the primary outcome as the most important while others left it outside their top 5.

## Discussion

In our sample of 44 mostly phase 3 trials with 46 primary outcomes, 29 patients, one patient representative and 12 healthcare professionals together ranked the primary outcome as the most important outcome 13/46 times or 28%. Given that so much hinges on the primary outcome even our small study should give some pause. Our respondents comprised people with lived experience of breast cancer or kidney disease and healthcare professionals who treat breast cancer or nephrology patients every day. In their collective view, trial teams got the choice of primary outcome wrong more often than they got it right.

This is a concern because, as the name suggests, the primary outcome is intended to be a trial’s most important outcome. It is so important that statisticians calculate how big the trial needs to be (i.e. the sample size) so as to be able to say something meaningful about the primary outcome results, something rarely done for other trial outcomes. Making a mistake in the choice of primary outcome could mean that the trial is too small to say something meaningful about what really matters to patients and healthcare professionals, or the trial could be bigger than it needs to be. The trial might say nothing at all about what matters most. The kindest thing that can be said about this is that it represents research waste. Less kindly, it means patients and healthcare staff have spent their time, energy, goodwill and perhaps hope on a trial that has failed to provide the key information that people like them need in order to make better treatment decisions. No doubt a lot of money has also been spent [9].

We are not the first to highlight this problem [3, 10, 11]. In 2017, Heneghan and colleagues wrote:

The treatment choices of patients and clinicians should ideally be informed by evidence that interventions improve patient-relevant outcomes. Too often, medical research falls short of this modest ideal [3].

Quite so. Our study shows that many primary outcomes are not the ones most important to patients and healthcare professionals, which they should be for late-stage trials such as phase 3 trials. Even phase 2 trials are generally done to inform a future phase 3 trial and outcomes generally reflect this. (As an aside, for nephrology where we had a mix of phase 2 and 3 trials, all five of the trials where our participants agreed with trial teams were phase 3 trials.) Most primary outcomes were in the top 5 ranking outcomes for a trial: they were important but not the *most* important. This is the critical thing about a primary outcome: if you are to nail your colours (and sample size) to a single outcome, then it *has* to be the one that matters most. ‘Quite important’ does not cut the mustard.

The solution is not difficult: ask people with lived experience of an illness or condition, and their healthcare professionals, what they want to know most. Funders, ethics committees and others involved in study approval have a role to play too. All should be asking to see researchers’ rationale for the choice of primary and other outcomes to ensure that the choices made are the right ones. We acknowledge that some preferred outcomes, survival say, can make trials long and potentially costly. Clearly, there needs to be a balance between what is desirable and what is possible. But the solution should not simply be to choose something else; there needs to be careful consideration of what might be lost and what second-best might be. Sometimes paying for what is desirable just might be worth it.

Core outcome sets — sets of outcomes already known to be important; see https://www.comet-initiative.org — have an important role because they are developed using formal methods of patient and other stakeholder involvement to choose outcomes [5]. It may still be necessary to decide which outcome in a core outcome set is *most* important but the people to decide that are patients/patient representatives and healthcare professionals, not researchers. Despite the availability of core outcome sets, 98% of trials do not use them, even when a relevant core outcome set exists [12]. Matvienko-Sikar and colleagues found that the most common barrier to the use of a core outcome set was trial team’s own outcome preferences [12] and as our work shows, those preferences do not always align with those of patients and healthcare professionals.

### Strengths and limitations

The key strength of our study is that we put real trial outcomes from two disease areas in front of people who have made, or are making, the sorts of decisions the trials were intended to support. There was an international mix to the patients and healthcare professionals involved and the number of trials they looked at (44) and the number of outcomes (273) are, we think, large enough to pay attention to. A larger number might have been better, but we know from the pilot nephrology study that our respondents would have baulked at more.

There are weaknesses too. There is no doubt that the task we gave patients, their representatives and healthcare professionals was a difficult one. Healthcare professionals contributing to the breast cancer work sometimes struggled with the short trial descriptions; six of the 14 comments from healthcare professionals mentioned this. These respondents were forced to make assumptions for these trials, which leads to uncertainty about their outcome ranking (and potentially that of others). One patient commented that the medical jargon for one trial was hard to understand. While we had no similar comments in the nephrology work, our pilot showed that our original 25-trial questionnaire was too time-consuming, which led us to reduce the number of trials any individual patient saw to five. This reduction, combined with FS and EM being present to answer questions, probably improved understanding in the nephrology work compared to our earlier breast cancer work.

We did not get public contributor comment on our outcome descriptions prior to using them with patients and their representatives in either the breast cancer or the nephrology work. We originally conceived the project, especially the breast cancer work, as stakeholder engagement work to improve future trials and the difficulty some participants may have had in understanding the outcomes is itself a finding. We are confident that the headline conclusion of this study is correct — that the outcome chosen as a primary outcome by trial teams is very often not the one most important to patients and health professionals. However, readers need to bear in mind that some respondents may not have fully understood some of the trials and/or some of the outcomes and that is clearly a limitation. That understanding trial outcomes can be hard work for healthcare professionals working in the field is something all of us who design trials should reflect on.

Outcomes, especially primary outcomes, are generally selected after discussion within a trial team, often including patients. Outcome decisions are therefore not normally made alone and having the opportunity to listen to others may change a person’s view of what should be measured. Our participants were not able to do this, which leaves open the possibility that agreement between participants and trial teams could have been different had the decisions been shared. Whether this difference would have been higher or lower is impossible to know. However, the median ranks shown in Tables 3 and 4 for the primary outcomes selected by trial teams are often well away from 1, the top ranked spot, suggesting that a lot of convincing would have been needed in those discussions to achieve agreement. As others have said [5–7], the selection of outcomes important to decision-makers is far from guaranteed.

We also invited people we knew: with more resources, larger groups and more open invitations would have given us greater confidence that the views expressed were representative. The breast cancer group had more health professionals, nephrology more patients and having groups with similar compositions would perhaps have been better. That said all patients and healthcare professionals deserved their place and we do not think that our headline result would change with different and/or bigger groups. It might change if we had chosen different clinical areas or chosen particular funders (see the ‘Implications for future research’ section).

We chose seven journals as the basis for our search, but a wider, non-journal-specific search would certainly have been more representative of trials in general. Trials reported in the *New England Journal of Medicine*, *The Lancet*, *BMJ* and *Journal of the American Medical Association* are likely to be from large, well-funded and experienced trial teams. Our view is that this means our results are likely to be conservative. Had we chosen trials published anywhere, we think the match between the primary outcome patients and health professionals want, and the primary outcome they got would be lower than the 28% we found.

### Implications for practice

- Trialists must consult with patients and healthcare professionals to identify the outcomes they will need to inform their future decisions about the usefulness of the intervention being tested. Trialists should ask them to rank these outcomes to avoid choosing the wrong primary outcome. Trialists should then resist requests to add to the outcome list without having a compelling reason for collecting data not essential to stakeholders’ future treatment decisions. Where a core outcome set [5] exists, trialists should use it.
- Understanding the outcomes presented in our selection of trials was sometimes hard not only for patients but for healthcare professionals with many years of experience. Trial teams should make sure their outcomes make sense to those expected to use them.
- Telling potential participants what the primary outcome is in participant information leaflets and trial recruitment discussions would help them to make better decisions as to whether the trial was measuring something they consider important and, therefore, whether the trial was something they should give their time to.

### Implications for future research

- It would be worth replicating our work in a few other clinical areas to see to what extent our findings are limited to breast cancer and nephrology or whether this represents a general problem. We think there is a general problem but knowing would be better than thinking. Replications would benefit from better trial descriptions than the very short ones we used in this study and from involving public contributors in writing outcome definitions. Having a researcher present to answer questions participants may have (as in our nephrology study) would be beneficial too.
- It would be worth exploring whether the situation is different for commissioned trials that give a primary outcome based on, for example, a James Lind Alliance Priority Setting Partnership (https://www.jla.nihr.ac.uk/about-the-james-lind-alliance/about-psps.htm) that has already involved consultation with stakeholders.

## Conclusion

Trials are done to improve decisions. To do this, trials need to be designed so that everything, from research question to dissemination, matches what those making decisions need. This includes outcome choice and especially that of the trial primary outcome. In our study, patients and healthcare professionals agreed with the choice of the primary outcome made by trial teams doing late-stage trials in breast cancer management and nephrology 28% of the time.

## Supplementary Information

**Additional file 1. **Search strategies. 

**Additional file 2.** An example of how two trials (both breast cancer), their outcomes and the short outcome definitions (where needed) were presented to participants.

**Additional file 3.** A participant information leaflet for the breast cancer study to send to the people we invited to stage 3.

**Additional file 4.** A patient information leaflet and signed an informed consent form.

**Additional file 5.** A summary of all 44 included trials. 

## Data Availability

The datasets used and/or analysed during the current study are available at https://osf.io/xkad6/.

## Abbreviations

MSc: Masters
