# Supplementary material for: Getting it wrong most of the time? Comparing trialists’ choice of primary outcome with what patients and health professionals want
Source: Trials. 2022 Jun 27;23:537. doi: 10.1186/s13063-022-06348-z (PMC9235090; doi:10.1186/s13063-022-06348-z)
Supplement: Supplementary file 1 — Additional file 1. Search strategies. [file 13063_2022_6348_MOESM1_ESM.pdf]

Search strategies

Breast Cancer Trials

Search 1: The BMJ Journal

(("breast neoplasms"[MeSH Terms] OR ("breast"[All Fields] AND "neoplasms"[All Fields]) OR "breast neoplasms"[All Fields]) AND ("randomized controlled trial"[Publication Type] OR "randomized controlled trials as topic"[MeSH Terms] OR "randomized controlled trial"[All Fields] OR "randomised controlled trial"[All Fields])) AND ("Br Med J"[Journal] OR "Br Med J (Clin Res Ed)"[Journal] OR "BMJ"[Journal] OR "bmj"[All Fields]) AND ("2015/01/01"[PDAT] : "2018/12/31"[PDAT])

Search 2: The Lancet Journal

(("breast neoplasms"[MeSH Terms] OR ("breast"[All Fields] AND "neoplasms"[All Fields]) OR "breast neoplasms"[All Fields]) AND ("randomized controlled trial"[Publication Type] OR "randomized controlled trials as topic"[MeSH Terms] OR "randomized controlled trial"[All Fields] OR "randomised controlled trial"[All Fields])) AND ("Lancet"[Journal] OR "Lancet Respir Med"[Journal] OR "Lancet Public Health"[Journal] OR "Lancet Psychiatry"[Journal] OR "Lancet Planet Health"[Journal] OR "Lancet Oncol"[Journal] OR "Lancet Neurol"[Journal] OR "Lancet Infect Dis"[Journal] OR "Lancet Haematol"[Journal] OR "Lancet HIV"[Journal] OR "Lancet Glob Health"[Journal] OR "Lancet Gastroenterol Hepatol"[Journal] OR "Lancet Diabetes Endocrinol"[Journal] OR "Lancet Child Adolesc Health"[Journal] OR "lancet"[All Fields]) AND ("2015/01/01"[PDAT] : "2018/12/31"[PDAT])

Search 3: New England Journal of Medicine (NEJM)

(("breast neoplasms"[MeSH Terms] OR ("breast"[All Fields] AND "neoplasms"[All Fields]) OR "breast neoplasms"[All Fields]) AND ("randomized controlled trial"[Publication Type] OR "randomized controlled trials as topic"[MeSH Terms] OR "randomized controlled trial"[All Fields] OR "randomised controlled trial"[All Fields])) AND ("N Engl J Med"[Journal] OR "nejm"[All Fields]) AND ("2015/01/01"[PDAT] : "2018/12/31"[PDAT])

Nephrology Trials

An initial search of Phase 3 trials in the journals, The Lancet and the New England Journal of Medicine revealed a small number of Phase 3 nephrology trials conducted in a five year period, so we expanded our search to include six high impact journals (Indexed in ISI Web of Science): The Lancet, The New England Journal of Medicine, The Journal of the American Medical Association, the Clinical Journal of the American Society of Nephrology, Kidney International and Nephrology Dialysis Transplantation; an expanded time frame and any of Phase 2 or 3 trials.

Search:
((((((((((((renal dialysis) OR (kidney disease)) OR (kidney injury)) OR (nephrotic syndrome)) OR (kidney transplant)) OR (haemodialysis)) OR (hemodialysis)) AND (randomised controlled trial)) OR (randomized controlled trial)) AND ("Lancet" [Journal])) OR ("N Engl J Med"[Journal])) OR ("JAMA"[Journal])) OR ("Clin J Am Soc Nephrol"[Journal])) OR ("Kidney Int"[Journal])) OR ("Nephrol Dial Transplant"[Journal]) Filters: Full text, Clinical Trial, Phase II, Clinical Trial, Phase III, Randomized Controlled Trial, from 2010 – 2020.
