# Supplementary material for: Getting it wrong most of the time? Comparing trialists’ choice of primary outcome with what patients and health professionals want
Source: Trials. 2022 Jun 27;23:537. doi: 10.1186/s13063-022-06348-z (PMC9235090; doi:10.1186/s13063-022-06348-z)
Supplement: Supplementary file 2 — Additional file 2. An example of how two trials (both breast cancer), their outcomes and the short outcome definitions (where needed) were presented to participants. [file 13063_2022_6348_MOESM2_ESM.pdf]

Trial outcomes: are the trial outcomes used in breast cancer research providing relevant information to support decision-making?

Article 1

A trial is testing the effects of a treatment with ribociclib (chemotherapy drug) plus letrozole (hormone therapy) compared to placebo plus letrozole as first-line treatment in postmenopausal women with positive hormone receptor (HR) and negative human epidermal growth factor receptor 2 (HER2). HR and HER2 influence the growth of the breast cancer cells.

If you were involved in a decision about the treatment above described, what would be the 5 most important things you would like to see measured in the trial (where 1 is the most important, 2 the next most important etc)? You do not need to choose 5 things if you don’t want to.

	1	2	3	4	5
**Safety.**	○	○	○	○	○
**Overall survival.**	○	○	○	○	○
**Overall response rate** (complete or partial response).	○	○	○	○	○
**Locally investigator-assessed progression-free survival.**	○	○	○	○	○
**Clinical benefit rate** (overall response plus stable disease lasting 24 weeks or more).	○	○	○	○	○
**Quality-of-life assessments.**	○	○	○	○	○

Please add and rank any other outcome that you think should have been measured but are not on the list.

Example: "My important outcome - rank 3"

Please share any comments you have.

Trial outcomes: are the trial outcomes used in breast cancer research providing relevant information to support decision-making?

Article 2

A trial is testing the clinical use of 70-gene signature test* compared with the standard clinical–pathological criteria in selecting patients for adjuvant chemotherapy.

*70-gene signature test is a genomic test developed to better predict clinical outcome and to determine whether the addition of adjuvant chemotherapy to endocrine therapy is worthwhile.

If you were involved in a decision about the use of the genomic test above described, what would be the 5 most important things you would like to see measured in the trial (where 1 is the most important, 2 the next most important etc)? You do not need to choose 5 things if you do not want to.

	1	2	3	4	5
**Overall survival** (time from trial randomisation until death from any cause).	○	○	○	○	○
**Survival without distant metastasis at 5 years** (time from trial randomisation until the first distant metastatic recurrence, or death from any cause).	○	○	○	○	○
**Disease-free survival** (time from trial randomisation until first disease progression or death from any cause).	○	○	○	○	○
**Proportion of patients who received chemotherapy based on the clinical risk as compared with the genomic risk.**	○	○	○	○	○

Please add and rank any other outcome that you think should have been measured but are not on the list.

Example: "My important outcome - rank 3"

Please share any comments you have.
