# Supplementary material for: Getting it wrong most of the time? Comparing trialists’ choice of primary outcome with what patients and health professionals want
Source: Trials. 2022 Jun 27;23:537. doi: 10.1186/s13063-022-06348-z (PMC9235090; doi:10.1186/s13063-022-06348-z)
Supplement: Supplementary file 3 — Additional file 3. A participant information leaflet for the breast cancer study to send to the people we invited to stage 3. [file 13063_2022_6348_MOESM3_ESM.pdf]

THE UNIVERSITY *of* EDINBURGH
MSc Clinical Trials

Trial outcomes: are the outcomes used in breast cancer trials providing the information needed to support decision-making?

The stakeholder panel - summary of activities

Introduction
Literature shows that there is huge inefficiency in trial conduct regarding the amount of data collected and its related costs[1]. Additionally, it has been reported that heterogeneity of outcome measurements can pose difficulties for interpretation[2].

Aim of the project
This project is intended to produce some evidence as to whether all the trial outcomes used in breast cancer trials are relevant for the individuals making decisions on whether to use the treatment or not.

Project highlights
Firstly, a list of trials designed to see if a treatment has important beneficial effects on breast cancer management and published from Jan-2015 to Dec-2018 will be selected from the BMJ, The Lancet and The New England Journal of Medicine (NEJM) journals. We will then randomly select 20 trials from this list. Secondly, we will create lists of the outcomes measured in the trials. We will present these lists to the members of our stakeholder panel and ask them to rank the five outcomes they would consider to be the most useful when making a decision about whether to use a treatment or not. The panel can add outcomes if they think something is missing. Finally, we will compare the rankings given by our panel members and that given by the trial teams. This will tell us something about how important the trial outcomes are to those using trial results to support treatment decisions.

Types of stakeholder on the panel
People who have, or have had, breast cancer; medical and clinical oncologists; surgeons; radiologists; breast care nurse; cancer organizations.

Panel members' activities
- Access the electronic survey (a link) with the list of outcomes extracted from the selected trials.
- Rank the five outcomes you think give the most useful and relevant information to support decision-making on whether to use a treatment or not.
- Provide any additional outcomes you would have liked to see but was not on the list. These outcomes can be part of your Top 5 outcomes.
- Let us know if there are any other type of stakeholder you think should be included in the panel and asked to rank the outcomes.

Considerations: 1. The survey is anonymous, and no personal data will be collected. 2. After closure of the survey, the overall responses from all users can be released upon request.

Timelines
If at all possible we would like the ranked responses back from panel members within two weeks of receiving the survey information. Survey expected to be distributed: Mid-April 2019.

THE UNIVERSITY *of* EDINBURGH
MSc Clinical Trials

Researcher

Viviane Miyakoda, MSc Clinical Trials' student of the University of Edinburgh with supervision of Prof. Shaun Treweek, Chair in Health Services Research of the University of Aberdeen.

References

1. KENNETH et al., (2018). Quantifying the Magnitude and Cost of Collecting Extraneous Protocol Data. American Journal of Therapeutics, 22, pp.117-124.
2. MCNAIR et al., (2016). Trial outcomes and information for clinical decision-making: a comparative study of opinions of health professionals. pp.(2016) Trial outcomes and information for clinical decision-making: a comparative study of opinions of health professionals. Trials, 17. 344.
