# Supplementary material for: Getting it wrong most of the time? Comparing trialists’ choice of primary outcome with what patients and health professionals want
Source: Trials. 2022 Jun 27;23:537. doi: 10.1186/s13063-022-06348-z (PMC9235090; doi:10.1186/s13063-022-06348-z)
Supplement: Supplementary file 4 — Additional file 4. A patient information leaflet and signed an informed consent form. [file 13063_2022_6348_MOESM4_ESM.pdf]

Title of study: Anonymous survey of patient and clinical staff opinions regarding the relevance of clinical trial outcomes regarding clinical decision making.

Principal Investigator:

Prof Joe Eustace, HRB Clinical Research Facility Cork, Tel: 021 4935143

Why am I being invited to participate in this study?

You are invited to participate in this study as you have experience in making clinical decisions in the area of nephrology (kidney disease) management either as a patient or as a health care professional.

Who is organising and running this study?

University College Cork are the sponsor of this study. It is being run by Professor Joe Eustace, Director of the HRB Clinical Research Facility Cork.

How do you select the participants?

We aim to recruit up to 12-15 participants consisting of healthcare professionals and patients in the clinical area of nephrology management.

Participant Entry Criteria

1. To have undergone treatment for nephrology in the last 10 years or are currently a healthcare professional involved in nephrology management.
2. To be willing to provide informed written consent and able to complete the survey.

What is the research focus?

The focus of the study is to examine the extent to which outcomes that are used in clinical trials are relevant to patient and clinicians who are involved in making clinical decisions.

Are there any benefits to taking part?

Completing this survey is unlikely to provide you with any direct benefit. You will not be paid for taking part. We hope that the study will help researchers to understand more about the relevance and usefulness of different clinical trial outcomes in helping to make clinical decisions. This will hopefully help to make future trials more responsive and relevant to the needs of patients and healthcare staff.

Are there any risks to taking part?

This study involves completing a survey only, so there are no known risks to taking part. No personal identifying data (e.g. Name, address etc.) about you will be collected.

What will I have to do?

If you agree to participate in this study, you will be given a survey by a member of the research team that will list the outcomes that were used in a range of different trials in the clinical area of nephrology management. For each trial the outcomes that were used will be presented in a random order. You will be asked to rank the outcomes for each trial in the order of importance from your view point (as either a patient or as a healthcare profession). We estimate that it will take you 20-25 minutes to complete the survey. No personal or identifying information is collected other than whether you are a patient or as a healthcare professional. There are no other subsequent follow-up activities.

Do I have to take part?

Participation is entirely voluntary, you can choose to take part or not. You can also change your mind and withdraw at any time. Whether you choose to take part or not will have no impact on your usual healthcare.

What about Data Protection?

All the study data is fully anonymised and no personal information identifying you or about you will be collected or stored.

Further information:

For more information or questions, feel free to contact the investigator:

Prof Joe Eustace, HRB Clinical Research Facility Cork, Mercy University Hospital, Grenville Place, Cork

Email j.eustace@ucc.ie,

Phone : 021 4935143

Title: Anonymous survey of patient and clinical staff opinions regarding the relevance of clinical trial outcomes regarding clinical decision making.

Consent Form

Principal Investigator's Name:
Professor Joe Eustace

Principal Investigator's Title:
Director, Health Research Board Clinical Research Facility, Cork

Principal Investigator's telephone No:
021 4935143

I confirm that the research project has been fully explained to me.

I have read and understood the Participant Information Leaflet V3.0 dated 21 Oct 2019 and have had an opportunity to ask questions about the project, to which I have had satisfactory answers.

I am aware that participation is ***voluntary*** and that I may withdraw my consent at any time without having to give a reason.

I am aware that my decision not to participate or to withdraw will not have any personal consequences for me or my ongoing care.

As the survey is anonymous I am aware that once I submit it, my answers are will no longer be identifiable and they cannot therefore be withdrawn.

I have received a copy of this consent form and the participant information leaflet for my records.

I, the undersigned, hereby consent to being a participant in the above described study conducted at the University College Cork. I understand that if I have any questions

concerning this research, I can contact the researcher listed above. If I have further queries concerning my rights in connection with the research, I can contact the Clinical Research Ethics Committee of the Cork Teaching Hospitals, Lancaster Hall, 6 Little Hanover Street, Cork.

*After reading the entire consent form, if you have no further questions about the study, please sign where indicated to give your consent.

....	....	....
Participant Signature	Name in Block Capitals	Date
....	....	....
Researcher Signature	Name in Block Capitals	Date
