# Supplementary material for: Getting it wrong most of the time? Comparing trialists’ choice of primary outcome with what patients and health professionals want
Source: Trials. 2022 Jun 27;23:537. doi: 10.1186/s13063-022-06348-z (PMC9235090; doi:10.1186/s13063-022-06348-z)
Supplement: Supplementary file 5 — Additional file 5. A summary of all 44 included trials. [file 13063_2022_6348_MOESM5_ESM.docx]

**Supplementary File 5**

**The 20 included breast cancer management trials**

| **Number** | **Title** | **Phase** |
| --- | --- | --- |
| 1 | Hortobagyi et al. Ribociclib as First-Line Therapy for HR-Positive, Advanced Breast Cancer. The New England Journal of Medicine 2016, 375(18), pp.1738–1748.  <https://www.nejm.org/doi/full/10.1056/nejmoa1609709> | 3 |
| 2 | Cardoso et al. 70-Gene Signature as an Aid to Treatment Decisions in Early-Stage Breast Cancer. The New England Journal of Medicine 2016, 375(8), pp.717–729.  <https://www.nejm.org/doi/10.1056/NEJMoa1602253> | 3 |
| 3 | Martin et al. Neratinib after trastuzumab-based adjuvant therapy in HER2-positive breast cancer (ExteNET): 5-year analysis of a randomised, double-blind, placebo-controlled, phase 3 trial. The Lancet Oncology 2017, 18(12), pp.1688–1700.  <https://www.thelancet.com/journals/lanonc/article/PIIS1470-2045(17)30717-9/fulltext> | 3 |
| 4 | Badwe et al. Locoregional treatment versus no treatment of the primary tumour in metastatic breast cancer: an open-label randomised controlled trial. The Lancet Oncology 2015, 16(13), pp.1380–1388.  <https://www.thelancet.com/journals/lanonc/article/PIIS1470-2045(15)00135-7/fulltext> | 3 |
| 5 | Polgár et al. Late side-effects and cosmetic results of accelerated partial breast irradiation with interstitial brachytherapy versus whole-breast irradiation after breast-conserving surgery for low-risk invasive and in-situ carcinoma of the female breast: 5-year results of a randomised, controlled, phase 3 trial. The Lancet Oncology 2017, 18(2), pp.259–268.  <https://www.thelancet.com/journals/lanonc/article/PIIS1470-2045(17)30011-6/fulltext> | 3 |
| 6 | Ganz et al. Patient-reported outcomes with anastrozole versus tamoxifen for postmenopausal patients with ductal carcinoma in situ treated with lumpectomy plus radiotherapy (NSABP B-35): a randomised, double-blind, phase 3 clinical trial. The Lancet 2016, 387(10021), pp.857–865.  <https://www.thelancet.com/journals/lancet/article/PIIS0140-6736(15)01169-1/fulltext> | 3 |
| 7 | Bear, H.D. et al. Neoadjuvant plus adjuvant bevacizumab in early breast cancer (NSABP B-40 NRG Oncology): secondary outcomes of a phase 3, randomised controlled trial. The Lancet. Oncology 2015, 16(9), pp.1037–1048.  <https://www.thelancet.com/journals/lanonc/article/PIIS1470-2045(15)00041-8/fulltext> | 3 |
| 8 | Del Mastro, L. et al. Fluorouracil and dose-dense chemotherapy in adjuvant treatment of patients with early-stage breast cancer: an open-label, 2 × 2 factorial, randomised phase 3 trial. Lancet 2015, 385(9980), pp.1863–1872.  <https://www.thelancet.com/journals/lancet/article/PIIS0140-6736(14)62048-1/fulltext> | 3 |
| 9 | Sparano, J.A. et al. Prospective Validation of a 21-Gene Expression Assay in Breast Cancer. The New England Journal of Medicine 2015, 373(21), pp.2005–2014.  <https://www.nejm.org/doi/10.1056/NEJMoa1510764?url_ver=Z39.88-2003&rfr_id=ori:rid:crossref.org&rfr_dat=cr_pub%3dwww.ncbi.nlm.nih.gov> | 3 |
| 10 | Robertson et al. Fulvestrant 500 mg versus anastrozole 1 mg for hormone receptor-positive advanced breast cancer (FALCON): an international, randomised, double-blind, phase 3 trial. The Lancet 2016, 388(10063), pp.2997–3005.  <https://www.thelancet.com/journals/lancet/article/PIIS0140-6736(16)32389-3/references> | 3 |
| 11 | Forbes, J.F. et al. Anastrozole versus tamoxifen for the prevention of locoregional and contralateral breast cancer in postmenopausal women with locally excised ductal carcinoma in situ (IBIS-II DCIS): a double-blind, randomised controlled trial. Lancet 2016, 387(10021), pp.866–73.  <https://www.thelancet.com/journals/lancet/article/PIIS0140-6736(15)01129-0/fulltext> | 3 |
| 12 | Zielinski et al. Bevacizumab plus paclitaxel versus bevacizumab plus capecitabine as first-line treatment for HER2-negative metastatic breast cancer (TURANDOT): primary endpoint results of a randomised, open-label, non-inferiority, phase 3 trial. The Lancet Oncology 2016, 17(9), pp.1230–1239.  <https://www.thelancet.com/journals/lanonc/article/PIIS1470-2045(16)30154-1/fulltext> | 3 |
| 13 | Krop et al. Trastuzumab emtansine versus treatment of physician's choice in patients with previously treated HER2-positive metastatic breast cancer (TH3RESA): final overall survival results from a randomised open-label phase 3 trial. The Lancet Oncology 2017, 18(6), pp.743–754.  <https://www.thelancet.com/journals/lanonc/article/PIIS1470-2045(17)30313-3/references> | 3 |
| 14 | Earl et al. Addition of gemcitabine to paclitaxel, epirubicin, and cyclophosphamide adjuvant chemotherapy for women with early-stage breast cancer (tAnGo): final 10-year follow-up of an open-label, randomised, phase 3 trial. The Lancet Oncology 2017, 18(6), pp.755–769.  <https://www.thelancet.com/journals/lanonc/article/PIIS1470-2045(17)30319-4/fulltext> | 3 |
| 15 | Earl et al. Efficacy of neoadjuvant bevacizumab added to docetaxel followed by fluorouracil, epirubicin, and cyclophosphamide, for women with HER2-negative early breast cancer (ARTemis): an open-label, randomised, phase 3 trial. The Lancet Oncology 2015, 16(6), pp.656–666.  <https://www.thelancet.com/journals/lanonc/article/PIIS1470-2045(15)70137-3/fulltext> | 3 |
| 16 | Baselga et al. Buparlisib plus fulvestrant versus placebo plus fulvestrant in postmenopausal, hormone receptor-positive, HER2-negative, advanced breast cancer (BELLE-2): a randomised, double-blind, placebo-controlled, phase 3 trial. The Lancet Oncology 2017, 18(7), pp.904–916.  <https://www.thelancet.com/journals/lanonc/article/PIIS1470-2045(17)30376-5/fulltext> | 3 |
| 17 | Cuzick et al. Tamoxifen for prevention of breast cancer: extended long-term follow-up of the IBIS-I breast cancer prevention trial. Lancet Oncology 2015, 16(1), pp.67–75.  <https://www.thelancet.com/journals/lanonc/article/PIIS1470-2045%2814%2971171-4/fulltext> | 3 |
| 18 | von Minckwitz, G. et al. Trastuzumab Emtansine for Residual Invasive HER2-Positive Breast Cancer. The New England Journal of Medicine 2019, 380(7), pp.617–628.  <https://www.nejm.org/doi/full/10.1056/NEJMoa1814017> | 3 |
| 19 | Finn, R.S. et al. Palbociclib and Letrozole in Advanced Breast Cancer. The New England Journal of Medicine 2016, 375(20), pp.1925–1936.  <https://www.nejm.org/doi/full/10.1056/NEJMoa1607303> | 3 |
| 20 | Poortmans, Philip M. et al. Internal Mammary and Medial Supraclavicular Irradiation in Breast Cancer. The New England Journal of Medicine 2015, 373(4), pp.317–327.  <https://www.nejm.org/doi/full/10.1056/nejmoa1415369> | 3 |

**The 24 included nephrology trials**

| **Number** | **Title** | **Phase** |
| --- | --- | --- |
| 1 | De Zeeuw, D., Akizawa, T., Audhya, P., Bakris, G.L., Chin, M., Christ-Schmidt, H., Goldsberry, A., Houser, M., Krauth, M., Lambers Heerspink, H.J. and McMurray, J.J., 2013. Bardoxolone methyl in type 2 diabetes and stage 4 chronic kidney disease. *New England Journal of Medicine*, *369*(26), pp.2492-2503. <https://nejm.org/doi/full/10.1056/NEJMoa1306033> | 3 |
| 2 | Macdougall, I.C., Provenzano, R., Sharma, A., Spinowitz, B.S., Schmidt, R.J., Pergola, P.E., Zabaneh, R.I., Tong-Starksen, S., Mayo, M.R., Tang, H. and Polu, K.R., 2013. Peginesatide for anemia in patients with chronic kidney disease not receiving dialysis. *New England Journal of Medicine*, *368*(4), pp.320-332. <https://www.nejm.org/doi/full/10.1056/NEJMoa1203166> | 3 |
| 3 | Cooper, C.J., Murphy, T.P., Cutlip, D.E., Jamerson, K., Henrich, W., Reid, D.M., Cohen, D.J., Matsumoto, A.H., Steffes, M., Jaff, M.R. and Prince, M.R., 2014. Stenting and medical therapy for atherosclerotic renal-artery stenosis. *New England Journal of Medicine*, *370*(1), pp.13-22. <https://www.nejm.org/doi/full/10.1056/NEJMoa1310753> | 3 |
| 4 | Agarwal, R., Rossignol, P., Romero, A., Garza, D., Mayo, M.R., Warren, S., Ma, J., White, W.B. and Williams, B., 2019. Patiromer versus placebo to enable spironolactone use in patients with resistant hypertension and chronic kidney disease (AMBER): a phase 2, randomised, double-blind, placebo-controlled trial. *The Lancet*, *394*(10208), pp.1540-1550. <https://www.thelancet.com/journals/lancet/article/PIIS0140-6736(19)32135-X/fulltext)> | 2 |
| 5 | Hutchison, C.A., Cockwell, P., Moroz, V., Bradwell, A.R., Fifer, L., Gillmore, J.D., Jesky, M.D., Storr, M., Wessels, J., Winearls, C.G. and Weisel, K., 2019. High cutoff versus high-flux haemodialysis for myeloma cast nephropathy in patients receiving bortezomib-based chemotherapy (EuLITE): a phase 2 randomised controlled trial. *The Lancet Haematology*, *6*(4), pp.e217-e228. <https://www.sciencedirect.com/science/article/abs/pii/S2352302619300146> | 2 |
| 6 | Jaber, S., Paugam, C., Futier, E., Lefrant, J.Y., Lasocki, S., Lescot, T., Pottecher, J., Demoule, A., Ferrandiere, M., Asehnoune, K. and Dellamonica, J., 2018. Sodium bicarbonate therapy for patients with severe metabolic acidaemia in the intensive care unit (BICAR-ICU): a multicentre, open-label, randomised controlled, phase 3 trial. *The Lancet*, *392*(10141), pp.31-40. https://www.sciencedirect.com/science/article/abs/pii/S0140673618310808 | 3 |
| 7 | Wilson, F.P., Shashaty, M., Testani, J., Aqeel, I., Borovskiy, Y., Ellenberg, S.S., Feldman, H.I., Fernandez, H., Gitelman, Y., Lin, J. and Negoianu, D., 2015. Automated, electronic alerts for acute kidney injury: a single-blind, parallel-group, randomised controlled trial. *The Lancet*, *385*(9981), pp.1966-1974. https://www.thelancet.com/journals/lancet/article/PIIS0140-6736(15)60266-5/fulltext | Unclear, but certainly not Phase 1 |
| 8 | Howman, A., Chapman, T.L., Langdon, M.M., Ferguson, C., Adu, D., Feehally, J., Gaskin, G.J., Jayne, D.R., O'Donoghue, D., Boulton-Jones, M. and Mathieson, P.W., 2013. Immunosuppression for progressive membranous nephropathy: a UK randomised controlled trial. *The Lancet*, *381*(9868), pp.744-751. <https://www.sciencedirect.com/science/article/pii/S0140673612615669> | Unclear, but certainly not Phase 1 |
| 9 | Iijima, K., Sako, M., Oba, M.S., Ito, S., Hataya, H., Tanaka, R., Ohwada, Y., Kamei, K., Ishikura, K., Yata, N. and Nozu, K., 2014. Cyclosporine C2 monitoring for the treatment of frequently relapsing nephrotic syndrome in children: a multicenter randomized phase II trial. *Clinical Journal of the American Society of Nephrology*, *9*(2), pp.271-278. <https://cjasn.asnjournals.org/content/9/2/271> | 2 |
| 10 | Ito, S., Shikata, K., Nangaku, M., Okuda, Y. and Sawanobori, T., 2019. Efficacy and safety of esaxerenone (CS-3150) for the treatment of type 2 diabetes with microalbuminuria: a randomized, double-blind, placebo-controlled, phase II trial. *Clinical Journal of the American Society of Nephrology*, *14*(8), pp.1161-1172. <https://cjasn.asnjournals.org/content/14/8/1161> | 2 |
| 11 | Ito, S., Kagawa, T., Saiki, T., Shimizu, K., Kuroda, S., Sano, Y. and Umeda, Y., 2019. Efficacy and safety of imarikiren in patients with type 2 diabetes and microalbuminuria: A randomized, controlled trial. *Clinical Journal of the American Society of Nephrology*, *14*(3), pp.354-363. <https://cjasn.asnjournals.org/content/14/3/354> | 2 |
| 12 | Tumlin, J., Goldman, J., Spiegel, D.M., Roer, D., Ntoso, K.A., Blaney, M., Jacobs, J., Gillespie, B.S. and Begelman, S.M., 2010. A phase III, randomized, double-blind, placebo-controlled study of tenecteplase for improvement of hemodialysis catheter function: TROPICS 3. *Clinical Journal of the American Society of Nephrology*, *5*(4), pp.631-636. <https://cjasn.asnjournals.org/content/5/4/631> | 3 |
| 13 | Rostaing, L., Massari, P., Garcia, V.D., Mancilla-Urrea, E., Nainan, G., del Carmen Rial, M., Steinberg, S., Vincenti, F., Shi, R., Di Russo, G. and Thomas, D., 2011. Switching from calcineurin inhibitor-based regimens to a belatacept-based regimen in renal transplant recipients: a randomized phase II study. *Clinical Journal of the American Society of Nephrology*, *6*(2), pp.430-439. <https://cjasn.asnjournals.org/content/6/2/430> | 2 |
| 14 | Bridoux, F., Carron, P.L., Pegourie, B., Alamartine, E., Augeul-Meunier, K., Karras, A., Joly, B., Peraldi, M.N., Arnulf, B., Vigneau, C. and Lamy, T., 2017. Effect of high-cutoff hemodialysis vs conventional hemodialysis on hemodialysis independence among patients with myeloma cast nephropathy: a randomized clinical trial. *Jama*, *318*(21), pp.2099-2110. <https://jamanetwork.com/journals/jama/fullarticle/2665000?resultClick=1> | 3 |
| 15 | Garg, A.X., Devereaux, P.J., Yusuf, S., Cuerden, M.S., Parikh, C.R., Coca, S.G., Walsh, M., Novick, R., Cook, R.J., Jain, A.R. and Pan, X., 2014. Kidney function after off-pump or on-pump coronary artery bypass graft surgery: a randomized clinical trial. *Jama*, *311*(21), pp.2191-2198. <https://jamanetwork.com/journals/jama/fullarticle/1877182?resultClick=1> | 3 |
| 16 | Carroll, J.K., Pulver, G., Dickinson, L.M., Pace, W.D., Vassalotti, J.A., Kimminau, K.S., Manning, B.K., Staton, E.W. and Fox, C.H., 2018. Effect of 2 clinical decision support strategies on chronic kidney disease outcomes in primary care: a cluster randomized trial. *JAMA network open*, *1*(6), pp.e183377-e183377. <https://jamanetwork.com/journals/jamanetworkopen/fullarticle/2709713?resultClick=1> | Unclear, but certainly not Phase 1 |
| 17 | Tuttle, K.R., Brosius III, F.C., Adler, S.G., Kretzler, M., Mehta, R.L., Tumlin, J.A., Tanaka, Y., Haneda, M., Liu, J., Silk, M.E. and Cardillo, T.E., 2018. JAK1/JAK2 inhibition by baricitinib in diabetic kidney disease: results from a Phase 2 randomized controlled clinical trial. *Nephrology Dialysis Transplantation*, *33*(11), pp.1950-1959. <https://academic.oup.com/ndt/article/33/11/1950/4903016?searchresult=1> | 2 |
| 18 | Yokoyama, K., Akiba, T., Fukagawa, M., Nakayama, M., Sawada, K., Kumagai, Y., Chertow, G.M. and Hirakata, H., 2014. A randomized trial of JTT-751 versus sevelamer hydrochloride in patients on hemodialysis. *Nephrology Dialysis Transplantation*, *29*(5), pp.1053-1060. <https://academic.oup.com/ndt/article/29/5/1053/1876199?searchresult=1> | 2 |
| 19 | Fishbane, S.N., Singh, A.K., Cournoyer, S.H., Jindal, K.K., Fanti, P., Guss, C.D., Lin, V.H., Pratt, R.D. and Gupta, A., 2015. Ferric pyrophosphate citrate (Triferic™) administration via the dialysate maintains hemoglobin and iron balance in chronic hemodialysis patients. *Nephrology Dialysis Transplantation*, *30*(12), pp.2019-2026. <https://academic.oup.com/ndt/article/30/12/2019/2460012?searchresult=1> | 3 |
| 20 | Haynes, R., and P. K. Judge. “Randomized Multicentre Pilot Study of Sacubitril/Valsartan versus Irbesartan in Patients with Chronic Kidney Disease: UK Heart and Renal Protection (UK HARP)-III. Rationale, Trial Design and Baseline Data.” Nephrology, Dialysis and Transplantation, vol. 32, no. 12, Oxford University Press, 2016, pp. 2043–51. <https://academic.oup.com/ndt/article/32/12/2043/3059462?searchresult=1> | Unclear, but certainly not Phase 1 |
| 21 | Hammer, F., Krane, V., Störk, S., Röser, C., Hofmann, K., Pollak, N., Allolio, B. and Wanner, C., 2014. Rationale and design of the mineralocorticoid receptor antagonists in end-stage renal disease study (MiREnDa). *Nephrology Dialysis Transplantation*, *29*(2), pp.400-405. <https://pubmed.ncbi.nlm.nih.gov/24166468/> | 2 |
| 22 | Qunibi, W.Y., Martinez, C., Smith, M., Benjamin, J., Mangione, A. and Roger, S.D., 2011. A randomized controlled trial comparing intravenous ferric carboxymaltose with oral iron for treatment of iron deficiency anaemia of non-dialysis-dependent chronic kidney disease patients. *Nephrology Dialysis Transplantation*, *26*(5), pp.1599-1607. <https://academic.oup.com/ndt/article/26/5/1599/1892199?searchresult=1>) | 3 |
| 23 | Ash, S.R., Singh, B., Lavin, P.T., Stavros, F. and Rasmussen, H.S., 2015. A phase 2 study on the treatment of hyperkalemia in patients with chronic kidney disease suggests that the selective potassium trap, ZS-9, is safe and efficient. *Kidney international*, *88*(2), pp.404-411. https://www.kidney-international.org/article/S2157-1716(15)32161-4/fulltext#s0045 | 2 |
| 24 | Rovin, B.H., Solomons, N., Pendergraft III, W.F., Dooley, M.A., Tumlin, J., Romero-Diaz, J., Lysenko, L., Navarra, S.V., Huizinga, R.B., Adzerikho, I. and Mikhailova, E., 2019. A randomized, controlled double-blind study comparing the efficacy and safety of dose-ranging voclosporin with placebo in achieving remission in patients with active lupus nephritis. *Kidney international*, *95*(1), pp.219-231. https://www.kidney-international.org/article/S0085-2538(18)30628-8/fulltext#sec3 | 2 |
